# Local intralesional talimogene laherparepvec therapy with complete local response in oral palatine mucosal melanoma: a case report

**DOI:** 10.1186/s13256-024-04554-8

**Published:** 2024-05-23

**Authors:** Saurabh D. Chitnis, Nolan B. Seim, Kari Kendra

**Affiliations:** 1 Division of Medical Oncology, The Ohio State University Comprehensive Cancer Center, Columbus, OH USA; 2 Department of Otolaryngology, The Ohio State University Wexner Medical Center, Columbus, OH USA

**Keywords:** Case report, Mucosal melanoma, Talimogene laherparepvec, T-VEC

## Abstract

**Background:**

Mucosal melanoma, an aggressive type of malignancy different from the cutaneous melanomas commonly seen in the head and neck region, represents < 1% of all malignant melanomas. The pathogenesis of mucosal melanoma is unknown. Targetable mutations commonly seen in cutaneous melanoma, such as in the *BRAF* and *NRAS* genes, have a lower incidence in mucosal melanoma. Mucosal melanoma carries a distinct mutational pattern from cutaneous melanoma. Surgery with negative margins is the first-line treatment for mucosal melanoma, and systemic therapy is not well defined. Talimogene laherparepvec, an oncolytic viral immunotherapy, is United States Food and Drug Administration approved for the treatment of advanced malignant cutaneous melanoma, with local therapeutic benefits. Mucosal melanoma was initially excluded from talimogene laherparepvec’s initial phase III clinical trial.

**Case presentation:**

We present the case of a white female patient in her 40s with past medical history of systemic lupus erythematous, scleroderma, and estrogen-receptor-positive invasive ductal breast carcinoma. Following a bilateral mastectomy, the patient was found to have *BRAF*-negative mucosal melanoma of her hard palate with a soft palate skip lesion. Owing to the presence of a skip mucosal lesion as well as the anticipated defect and need for free-flap reconstructive surgery, nonsurgical management was considered. The patient was referred to medical oncology, where—based on the patient’s complicated medical history and the risk of immunotherapy possibly worsening her prior autoimmune diseases—local talimogene laherparepvec injections were chosen as the primary therapy for her mucosal lesions. Though talimogene laherparepvec is approved for the treatment of cutaneous melanoma, there are limited data available on the use of talimogene laherparepvec in mucosal melanomas.

**Conclusion:**

The patient had a complete local tumor response at both the primary lesion as well as the skip lesion with the local injections. She had no side effects and maintained a high quality of life during treatment.

## Background

Melanomas are characterized as malignant cancers appearing from melanocytes commonly localized to the basal layer of the epidermis; these give rise to cutaneous melanomas that comprise the main bulk of the disease. Melanocytes have also been noted at extracutaneous sites such as the eyes, mucosal tissue, and leptomeninges. On rare occasions, melanomas can also arise from these melanocytes. Mucosal melanomas (MMs) develop in mucosal membranes lining the respiratory, gastrointestinal, and genitourinary tracts. Though they occur from the same cell type, MMs are an aggressive malignancy with a 5-year survival rate of only 25%, amplified by late diagnosis due to the obscure site of origin, mucosal tissue, with its rich vascular and lymphatic features [1].

Though surgery with wide negative margins is considered the first-line treatment for MM, this recommendation is only based on prior case series. Owing to the rarity of the disease, there are very few to no prospective studies on MM to suggest improved outcomes and better long-term survival with an aggressive surgical approach [1–3]. Surgery and possible adjuvant therapy, with either systemic immunotherapy or targeted therapy, are still considered the treatment of choice in cutaneous melanoma, with good prognosis depending on the stage of the disease, *BRAF* mutation status, and risk of recurrence [4]. Neoadjuvant immunotherapy is still under investigation for cutaneous melanoma [4]. MM, though known to arise from the same melanocytes, does not carry the same genetic mutations as its cutaneous counterpart and is not eligible for treatment with currently available targeted therapies. Whole-genome and whole-exome sequencing of MM have shown striking contrasts between mucosal and cutaneous melanomas. MM is known to have a higher mutational burden, so the benefits of targeted therapy and immunotherapeutic approaches are still being investigated [5].

Talimogene laherparepvec (T-VEC) is an oncolytic virus developed with additional immunotherapeutic properties, approved by the US Food and Drug Administration (FDA) in October 2015 to treat unresectable metastatic stage IIIB/C–IVM1a melanoma with cutaneous, subcutaneous, or nodal metastases. T-VEC is an injectable oncolytic herpes simplex-1 virus (HSV-1) that is genetically engineered to replicate in tumor cells, promoting tumor lysis and antitumor immune response [6–8]. The virus is also genetically modified to generate granulocyte–macrophage colony stimulating factor (GM-CSF), which can stimulate dendritic cells to produce a systemic antitumor response. Though clinical trials in cutaneous melanoma have shown significant response with a mild toxicity profile, T-VEC has not been specifically studied in mucosal melanoma.

We present a case where T-VEC was used to treat mucosal melanoma in a patient not eligible for other anticancer systemic therapies. The patient demonstrated a complete local tumor response, including a skip mucosal lesion, while preserving a high quality of life without significant treatment side effects.

## Case presentation

**Fig. 1:**
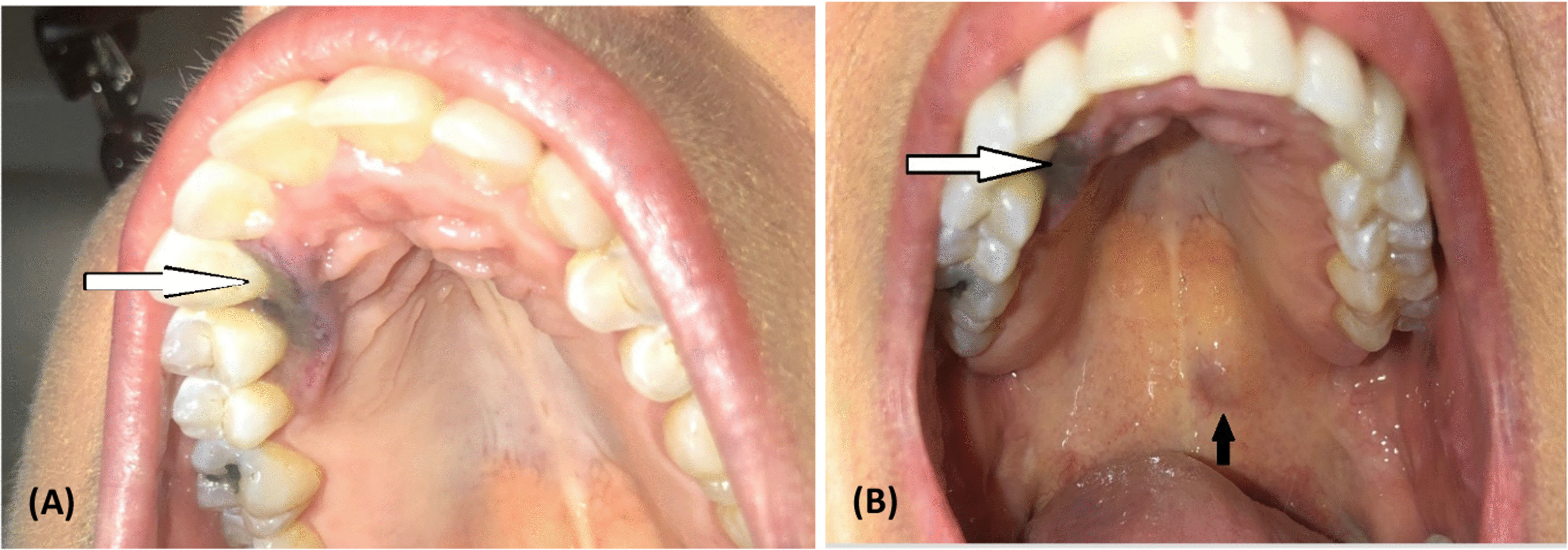
**A**, **B** Pictures from the initial evaluation. White arrows indicate the hard palate lesion, while the black arrow points to the soft palate lesion

A 47-year-old white female patient with known prior history of estrogen-receptor-positive invasive ductal carcinoma of the right breast, post-bilateral mastectomy, and prolonged history of systemic lupus erythematous (SLE) and scleroderma on long-term therapy with hydroxychloroquine presented to the head and neck surgery (ENT) clinic after a biopsy from the right anterior maxilla was positive for invasive melanoma. The patient’s dentist first noted a lesion on her right hard palate 4 months prior to initial evaluation, but it was not biopsied at that time. The lesion continued to grow over the next few months, and a biopsy was obtained. Initial pathology showed a Breslow depth of at least 1.2 mm with no ulceration and margins positive for malignancy. The *BRAF* was wild type. A clinical examination showed a dark, discolored lesion on the hard palate from the right canine to the second premolar tooth, with some extension to the labial surface and gingiva, measuring about 1.5 × 0.5 cm^2^ (Fig. 1A). Another small, approximately < 1-cm area of submucosal fullness with dark pigmentation was identified on the posterior left soft palate, just posterior to the hard palate junction (Fig. 1B). A flexible fiberoptic laryngoscopy on initial evaluation was negative for further local spread in the nasal cavity, nasopharynx, and oropharynx; no additional gross mass or lesion was seen, and there was no evidence of invasion on the nasal cavity from either lesion.

A positron emission tomography (PET) computed tomography (CT) scan showed avidity at both lesions. The left soft palate hypermetabolic focus had a maximum standardized uptake value (SUV) of 6.5, but no evidence of other suspicious active or distant metastasis was seen. Magnetic resonance imaging (MRI) of the brain showed no metastases, and facial MRI images identified the right hard palate and left soft palate lesions with no further local extension of the masses. The options for treatment were reviewed with the patient. Surgical treatment would have involved a right infrastructure maxillectomy and a separate soft palate resection with free-flap reconstruction and a possible obturator for the maxilla. The possible short- and long-term effects of surgery were reviewed with the patient with the primary concern for post-surgical velopharyngeal insufficiency. The case was reviewed at our multidisciplinary tumor board with the recommendation to proceed with systemic therapy in a primary versus neoadjuvant format given the presence of a mucosal skip lesion and the risk of future metastatic disease.

**Fig. 2:**
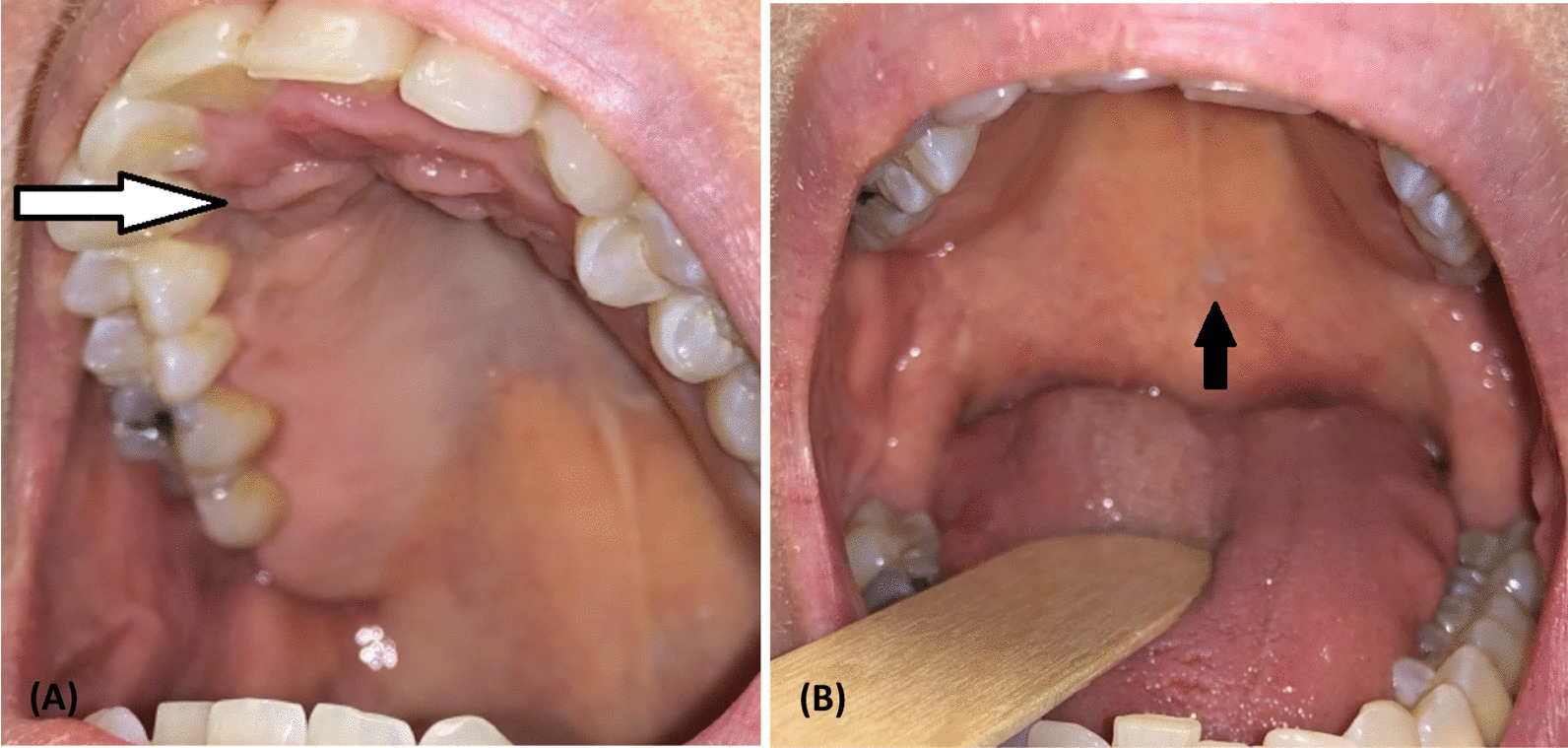
**A**, **B **Pictures from post-treatment evaluation. **A** The white arrow indicates the location of the hard palate lesion. **B** The black arrow points to the scar tissue of the soft palate lesion

The patient was offered chemoradiation with paclitaxel or intratumoral injection therapy with T-VEC. She elected to proceed with T-VEC owing to the advantages of local response with minimal risk for distant toxicity. The patient tolerated the initial test dose of 0.5 ml of 10^6^ plaque-forming units (PFUs) intralesionally well and proceeded per standard protocol to the recommended intralesional treatment dose of 1 ml of 10^8^ PFUs every 2 weeks. The total dose of 1 ml of 10^8^ PFUs was split between the two oral lesions. The goal for treatment was to continue T-VEC injections to the hard and soft palate lesions until there was no further detectable disease. The soft palate lesion regressed after 4 months of every-other-week T-VEC injections; a small subcentimeter scar without discoloration appeared at the site. A small new discolored lesion was noted on the right labial gingiva next to the last molar around 7 months after treatments were started but regressed with a single T-VEC injection at that site (lesion never biopsied). Another new lesion at the right soft palate at the superior tonsillar pillar appeared 10 months after the start of initial treatment; this was treated with T-VEC injections and resolved in just over 1 month (lesion never biopsied). The patient had an excellent response in the hard palate lesion after 21 cycles with no new lesions noted (Fig. 2). A PET CT scan repeated just over 1 year after her initial examination showed interval resolution of the hypermetabolic foci with no new suspicious lesions. Repeat biopsies of both the maxilla and soft palate lesions showed no evidence of active disease with hyperplastic mucosa with inflammation and scar tissue. Further treatments were held, and the patient was transitioned to active surveillance having received 21 cycles of T-VEC injections over a 10-month period.

**Fig. 3:**
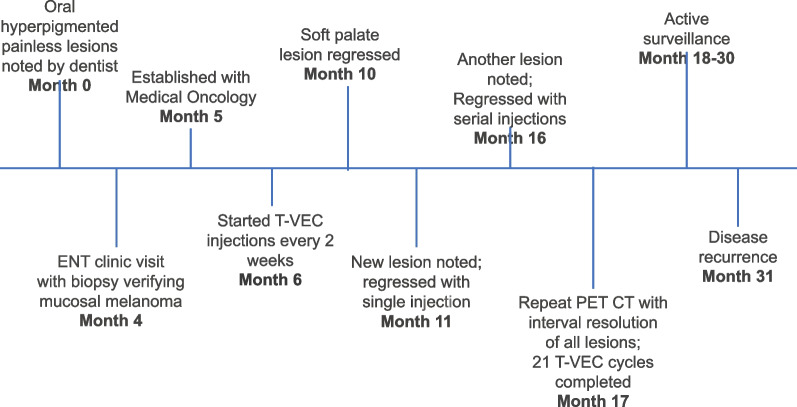
Case timeline

The patient remained disease-free for 1 year off treatment, but then developed new metastatic disease in her right adrenal gland and small bowel on repeat PET CT scans as described in the attached timeline (Fig. 3). She required surgical resection and adjuvant immunotherapy after approval from her rheumatologist. She remained locally disease free throughout, with her oropharynx intact, and continues today.

## Discussion and conclusion

For all melanoma diagnoses, the incidence of mucosal melanoma is 1.3% compared with 91.2% for cutaneous melanoma [9]. Head and neck MM has an incidence of 55.4% of all MM cases [9]. Owing to the low incidence of the disease and limited MM data, MM treatment regimens are still based on cases of the more common cutaneous melanoma. This case report, presenting a patient with hard and soft palate MM who was treated with T-VEC injections, is unique as T-VEC has not been specifically investigated for MM management. The OPTiM study, a randomized phase III trial of T-VEC versus GM-CSF, was designed to assess response in unresected melanoma with regional or distant metastases with injectable, subcutaneous, or nodal lesions present [10]. This trial assessed only cutaneous melanoma and did not include patients with MM.

A review of current published data reveals only case reports where T-VEC was used to treat MM. Our case is distinctive as only T-VEC therapy was used without adjunctive immunotherapy. Fröhlich *et al.* describe a patient with genitourinary MM who was initially treated with pembrolizumab-based immunotherapy followed by local T-VEC injections owing to locoregional disease progression. This patient showed initial improvement in local disease but was noted to have subsequent progression of nodal disease requiring immunotherapy resumption [11]. Drescher *et al.* reported on a patient with maxillary sinus mucosal melanoma where T-VEC injections were utilized to treat disease recurrence post surgical resection and radiation therapy while on maintenance immunotherapy with nivolumab [12]. Though both patients displayed significant response in local disease with the T-VEC injections, the patients had also received immunotherapy preceding the T-VEC therapy. Our reported case differs from these cases as the patient did not have metastatic disease nor surgical intervention or radiation therapy and was considered high risk for immunotherapy given her underlying rheumatological diseases. Our patient achieved complete locoregional response with progression-free survival of 1 year with exclusive T-VEC therapy without any adjunctive immunotherapy and remains disease free in the head and neck today. T-VEC intralesional injections in local MM need to be studied further to assess whether our findings can be supported in a randomized clinical trial.

Combined therapy using T-VEC with immunotherapy is currently being investigated in various clinical trials. The NIVEC trial is studying the effect of neoadjuvant nivolumab plus T-VEC therapy for resectable early-stage or metastatic cutaneous melanoma with injectable sites [13]. A randomized phase II trial assessing neoadjuvant T-VEC plus surgery versus surgery alone is also currently in process for resectable cutaneous stage IIIB–IVM1a melanoma [14]. Neoadjuvant T-VEC therapy is also being considered in the treatment of other malignancies, such as triple-negative breast cancer treated with neoadjuvant chemotherapy.

The fact that the mutational profile of MM is different from cutaneous melanoma requires further insight and research to guide future therapeutics [5]. Surgical resection with negative margins remains the primary therapy for resectable MM. Immunotherapy with anti-CTLA4 and anti-PD-1 agents has not been studied in MM in a large clinical trial, and response rates in MM have been observed to be lower than in cutaneous melanoma [11]. Radiation therapy for locoregional control is also being evaluated, though it is not known to improve long-term survival and has significant side effects.

Since the landscape for melanoma treatment has seen significant advances in recent years with immune-modulating therapeutics, T-VEC intralesional injections should be considered as an important tool for managing this disease and warrant further investigation.

## Data Availability

Not applicable.

## Abbreviations

CTLA4: Cytotoxic T-lymphocyte-associated protein 4
ENT: Otolaryngology
FDA: United States Food and Drug Administration
GM-CSF: Granulocyte–macrophage colony stimulating factor
MM: Mucosal melanoma
PD-1: Programmed cell death protein 1
PFUs: Plaque-forming units
SLE: Systemic lupus erythematous
SUV: Standardized uptake value
T-VEC: Talimogene laherparepvec

## References

[1] Olla D, Neumeister MW (2021). Mucosal melanoma. Clin Plast Surg.

[2] Yde SS, Sjoegren P, Heje M, Stolle LB (2018). Mucosal melanoma: a literature review. Curr Oncol Rep.

[3] Jeong YJ, Thompson JF, Ch’ng S (2023). Epidemiology, staging and management of mucosal melanoma of the head and neck: a narrative review. Chin Clin Oncol.

[4] Kobeissi I, Tarhini AA (2022). Systemic adjuvant therapy for high-risk cutaneous melanoma. Ther Adv Med Oncol.

[5] Nassar KW, Tan AC (2020). The mutational landscape of mucosal melanoma. Semin Cancer Biol.

[6] Fukuhara H, Ino Y, Todo T (2016). Oncolytic virus therapy: a new era of cancer treatment at dawn. Cancer Sci.

[7] Ferrucci PF, Pala L, Conforti F, Cocorocchio E (2021). Talimogene laherparepvec (T-VEC): an intralesional cancer immunotherapy for advanced melanoma. Cancers.

[8] Bommareddy PK, Patel A, Hossain S, Kaufman HL (2017). Talimogene laherparepvec (T-VEC) and other oncolytic viruses for the treatment of melanoma. Am J Clin Dermatol.

[9] Chang AE, Karnell LH, Menck HR. The National Cancer Data Base report on cutaneous and noncutaneous melanoma: a summary of 84,836 cases from the past decade. The American College of Surgeons Commission on Cancer and the American Cancer Society. Cancer. 1998;83(8):1664–78. 10.1002/(sici)1097-0142(19981015)83:8<1664::aid-cncr23>3.0.co;2-g 9781962

[10] Andtbacka RHI, Agarwala SS, Ollila DW, Hallmeyer S, Milhem M, Amatruda T (2016). Cutaneous head and neck melanoma in OPTiM, a randomized phase 3 trial of talimogene laherparepvec versus granulocyte-macrophage colony-stimulating factor for the treatment of unresected stage IIIB/IIIC/IV melanoma. Head Neck.

[11] Fröhlich A, Hoffmann F, Niebel D, Egger E, Kukuk GM, Toma M (2020). Talimogene laherparepvec in advanced mucosal melanoma of the urethra upon primary resistance on immune checkpoint inhibition: a case report. Front Oncol.

[12] Drescher C, Drexler K, Hilber H, Schmidt B, Haferkamp S (2019). Regression of mucosal melanoma following intralesional talimogene laherparepvec (T-VEC) injection in combination with immunotherapy. J Dtsch Dermatol Ges J Ger Soc Dermatol JDDG.

[13] Rohaan MW, Stahlie EHA, Franke V, Zijlker LP, Wilgenhof S, van der Noort V (2022). Neoadjuvant nivolumab + T-VEC combination therapy for resectable early stage or metastatic (IIIB-IVM1a) melanoma with injectable disease: study protocol of the NIVEC trial. BMC Cancer.

[14] Dummer R, Gyorki DE, Hyngstrom J, Berger AC, Conry R, Demidov L (2021). Neoadjuvant talimogene laherparepvec plus surgery versus surgery alone for resectable stage IIIB–IVM1a melanoma: a randomized, open-label, phase 2 trial. Nat Med.

